# A narrative review of functional near-infrared spectroscopy (fNIRS) applications in hearing loss, tinnitus and vestibular disorders

**DOI:** 10.3389/fnins.2025.1703917

**Published:** 2026-01-23

**Authors:** Davide Brotto, Gaia Lucarini, Valeria Del Vecchio, Nicole Galoforo, Elisa Lovato, Benedetta Colavolpe, Giusy Melcarne, Gino Marioni, Judit Gervain, Anna Rita Fetoni, Patrizia Trevisi

**Affiliations:** 1 Section of Otolaryngology, Department of Neuroscience DNS, University of Padova, Padova, Italy; 2 Department of Developmental and Social Psychology, University of Padova, Padova, Italy; 3 Padova Neuroscience Center, University of Padova, Padova, Italy; 4 Hearing and Balance Unit, Department of Head and Neck, Federico II University Hospital, Naples, Italy; 5 Phoniatrics and Audiology Unit, Department of Neuroscience DNS, University of Padova, Treviso, Italy; 6 Integrative Neuroscience and Cognition Center, Université Paris Cité & CNRS, Paris, France; 7 Department of Neuroscience and Reproductive Sciences and Dentistry, Audiology Section, University of Naples Federico II, Naples, Italy

**Keywords:** cochlear implant, fNIRS, hearing loss, tinnitus, vertigo

## Abstract

**Introduction:**

Functional near-infrared spectroscopy (fNIRS) has emerged as a promising neuroimaging modality for investigating cortical activity in auditory and vestibular domains. Its portability, device compatibility, and motion tolerance make it particularly suited for use in populations that are challenging to study with conventional neuroimaging techniques, such as infants and cochlear implant (CI) users. The present study aims to explore the potential and limitations of this neuroimaging technique in the audiological and vestibular fields, offering an integrated perspective across pediatric, adult and elderly populations.

**Methods:**

A narrative review of studies using fNIRS in hearing loss, tinnitus, and vestibular disorders was conducted through searches in PubMed and Scopus up to March 2025. Studies were included if they employed fNIRS to investigate cortical responses in individuals with diagnosed hearing loss, chronic tinnitus or to investigate vestibular function.

**Results:**

A total of 60 studies were reviewed: 36 on hearing loss, 11 on tinnitus, and 13 on vestibular disorders. In hearing research, fNIRS successfully identified cortical activation patterns related to auditory perception, speech processing, and cross-modal plasticity in CI users across development, adulthood and aging. The technique showed prognostic potential in predicting CI outcomes and monitoring listening effort and cognitive load. In tinnitus research, fNIRS consistently demonstrate hyper-activation in the auditory cortex and altered functional connectivity with frontal-limbic networks, reflecting sensory, cognitive, and emotional involvement. The technique was sensitive to treatment effects following interventions such as transcranial stimulation, acupuncture, and cochlear implantation. In vestibular research, fNIRS enabled the mapping of cortical networks involved in balance control and multisensory integration during various stimulation paradigms, including caloric testing, motion platforms, and optic flow in virtual environments. Although current applications are mostly exploratory, findings suggest fNIRS can capture vestibular-related cortical activity in real-world conditions.

**Conclusion:**

fNIRS offers a valuable, non-invasive, and ecologically valid method for investigating auditory and vestibular function across the lifespan. In hearing and tinnitus research, it shows strong potential for clinical translation, especially if methodological standardization is achieved. Applications in vestibular research remain preliminary but promising.

## 1 Introduction

**Figure 1:**
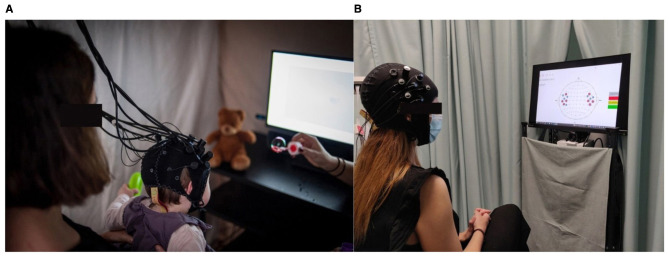
Different NIRS setups and participants from infancy **(A)** to adulthood **(B)**.

Understanding how the human brain processes auditory and vestibular information is central to neuroscience, audiology, and vestibular research. Although peripheral mechanisms underlying hearing and balance are increasingly well defined, the cortical correlates of auditory perception, hearing loss, tinnitus, and vestibular integration remain partially unknown. In recent years, functional near-infrared spectroscopy (fNIRS) has gained attention as a promising neuroimaging tool to explore these aspects, offering advantages such as portability, silent acquisition, and resilience to motion artifacts—features particularly relevant in auditory and vestibular research contexts-and allowing its use from early childhood through older adulthood (Anderson et al., 2019; Nguyen et al., 2020; Shoushtarian et al., 2024) (Figure 1).

Unlike other neuroimaging modalities (e.g., functional magnetic resonance imaging, fMRI or proton emission tomography, PET), fNIRS allows the study of populations traditionally underrepresented in cortical functional research, such as infants, cochlear implant (CI) users, and individuals with balance disorders (Rovetti et al., 2019; Saksida et al., 2021; Lee et al., 2023), for whom other diagnostic techniques are challenging to apply (due to radiation exposure or the need for sedation) or may be limited by artifacts, as often occurs in children or in noisy environments. Furthermore, this technique can be used from early childhood, without interfering with children's ability to perform simple tasks, and is harmless even when repeated several times, thereby enabling objective comparison in longitudinal studies.

**Figure 2:**
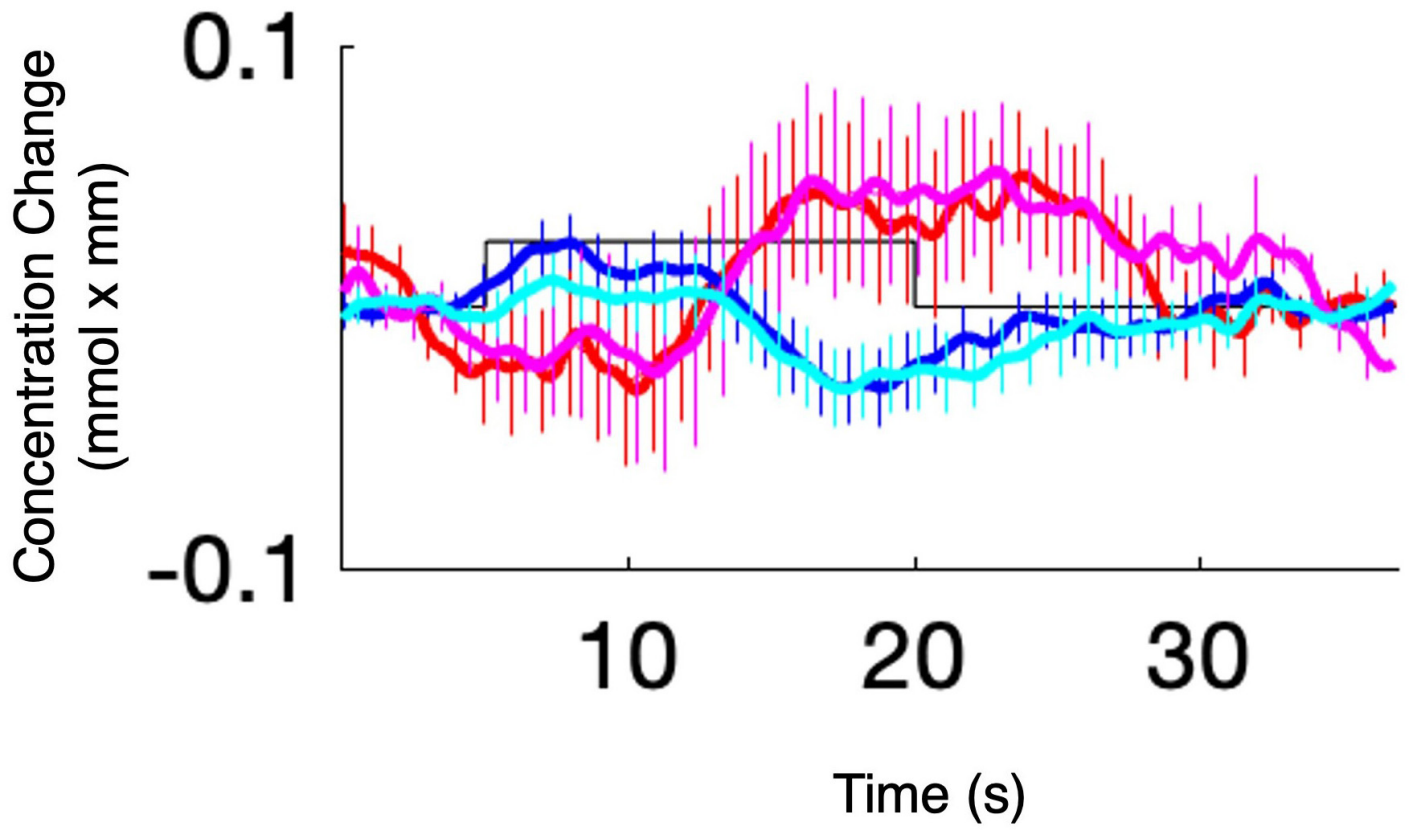
A typical hemodynamic response function in a group of 6-month-old infants.

Several studies have demonstrated that fNIRS can detect task-dependent and resting-state cortical activation in response to acoustic stimuli (Balint et al., 2024) (Figure 2), to hearing restoration via hearing aids or CIs (Harrison et al., 2021; Zhou et al., 2022), and to vestibular stimulation in both healthy individuals and patients with visual vertigo or vestibular dysfunction (Hoppes et al., 2018a; Valdés et al., 2021). In tinnitus research, fNIRS has enabled the observation of cortical plasticity and treatment effects through interventions such as acupuncture, neuromodulation, or cochlear implantation (Verma et al., 2019; Yu et al., 2023; Hu et al., 2024).

Nonetheless, methodological challenges persist. fNIRS presents: (1) reduced spatial resolution compared to fMRI, (Lloyd-Fox et al., 2010); (2) limited depth sensitivity, restricting analysis to superficial cortical layers, with age-related variation, making the technique particularly suitable and effective for young children, (Beauchamp et al., 2011); (3) temporal resolution that may limit its use in studying temporal features critical to auditory processing; and (4) cortical coverage dependent on probe design and optode density, with different configurations offering varying level of spatial sensitivity (Yücel et al., 2021). Moreover, anatomical variables, such as the skull thickness and scalp-to-cortex distance, may affect signal quality and interpretation, especially in pediatric or aging populations (Chen et al., 2016; Liu et al., 2023). These factors are particularly relevant when investigating subcortical auditory and vestibular neural structures, which may play a central role in symptom generation.

In this narrative review, recent literature on the application of fNIRS in the study of hearing and balance has been analyzed. Three key areas are addressed: (1) cortical responses to sound stimulation in hearing-impaired populations, (2) neural reorganization and treatment monitoring in tinnitus; and (3) cortical responses to vestibular stimulation. By analyzing study populations, experimental paradigms, and methodological approaches, we aim to delineate the current role of fNIRS and discuss its translational implications for neuroscience, audiology, and vestibular research.

Most articles presented in literature provide different approaches to the abovementioned topics, thus impairing the chance of a systematic revision. Additionally, fNIRS application in tinnitus patients and to study vestibular function is still pioneering, with few studies available. For these reasons, we opted to address these topics proposing a narrative review.

## 2 Material and methods

A literature search was conducted using the PubMed and Scopus databases with the keywords “fNIRS,” “functional near-infrared spectroscopy,” AND (“tinnitus” OR “hearing loss” OR “hearing research” OR “vertigo” OR “balance disorders” OR “vestibular system”). Peer-reviewed articles published up to March 2025 were included if they were written in English, and used fNIRS as a primary or integrated method to investigate brain activity in individuals diagnosed with hearing loss, subjective tinnitus, or vertigo/balance disorders. Exclusion criteria were studies involving animal models, reviews, papers in which fNIRS was not specifically used for the abovementioned clinical disorders or protocols without data collection.

## 3 Results

Following a screening based on titles and abstracts, and the removal of duplicates, a total of 36 studies on hearing loss, 11 studies on tinnitus, and 13 on the vestibular disorders were retained.

**Figure 3:**
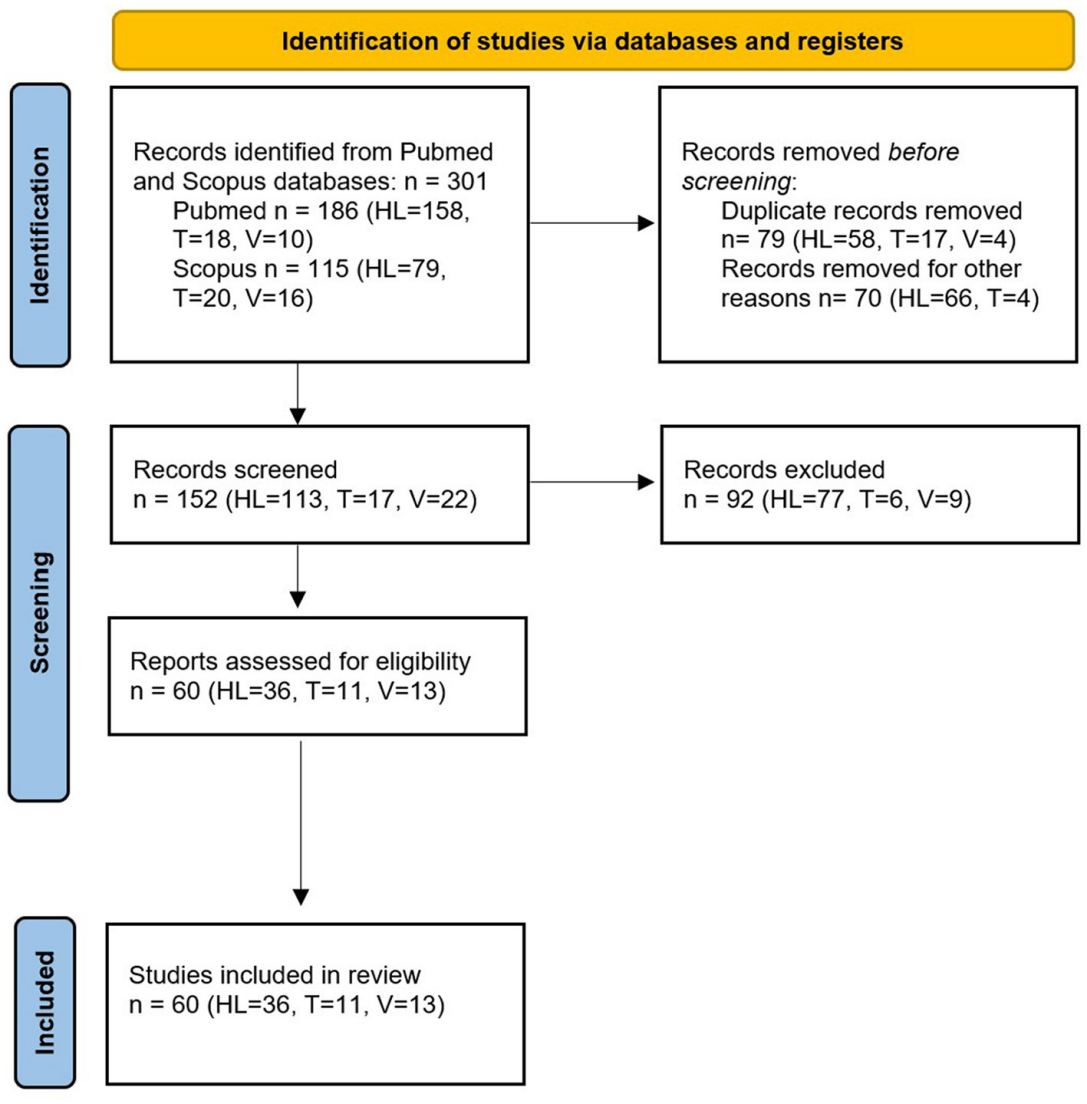
PRISMA diagram summarizing the electronic database search and inclusion/exclusion process of the review.

The summary of the selection process is shown in the Preferred Reporting Items for Systematic Reviews and Meta-Analyses (PRISMA) diagram (Figure 3). The main characteristics of the included papers are reported in Supplementary Tables 1–3.

## 4 Discussion

### 4.1 fNIRS applications in hearing disorders

Hearing disorders can lead to abnormal development of the central nervous system when the impairment is congenital, as is often the case in children, and may also result in the degeneration of the same neural structures when the auditory disability is acquired. Consequently, studying these neural structures is crucial to assess the impact of hearing loss and rehabilitative interventions (i.e., hearing aids and CIs) on their development, with the aim of understanding whether suboptimal results may be due to insufficient peripheral stimulation or to inadequate organization of central auditory pathways.

The specific features that make fNIRS a promising tool in this field are: (1) its compatibility with hearing devices, including CIs, (2) its tolerance to motion, which is particularly useful for infants or toddlers, who are usually less cooperative, and (3) its silent operation, which makes it especially suitable for studying auditory function across diverse populations. For all these reasons, fNIRS is more practical and suitable than fMRI for studies in young children with hearing loss.

#### 4.1.1 Study populations and design

fNIRS has been employed across a wide range of participant groups from infants to the elderly, with sample sizes varying substantially depending on study design and population, ranging from small cohorts of 9 participants (Dewey and Hartley, 2015; Bisconti et al., 2016; Sheffield et al., 2023) to larger cross-sectional studies involving more than 80 subjects (Liu et al., 2023, 2025; Zhou et al., 2023; Tan et al., 2024; Wang et al., 2025; Wu et al., 2025). Specifically, sample sizes are comparable across adult and infant studies. Adult studies report sample sizes ranging between 10 (Bisconti et al., 2016; Mai et al., 2024) to 63 (Lee et al., 2025) participants, while infant studies include between 9 (Coëz et al., 2022) and 84 (Wu et al., 2025) participants (see also Supplementary Table 1).

In pediatric populations, fNIRS has been used to assess cross-modal plasticity (Mushtaq et al., 2020; Wang et al., 2022; Liu et al., 2023, 2025; Deroche et al., 2024; Tan et al., 2024), speech perception (Chen et al., 2022; Coëz et al., 2022), auditory processing (Sevy et al., 2010), and cognitive effort (Balint et al., 2024). These studies usually include prelingually deaf children with CIs, often implanted due to congenital causes (Deroche et al., 2024; Wu et al., 2024, 2025) such as genetic syndromes or infections (e.g., congenital toxoplasmosis (Bertachini et al., 2021; Wang et al., 2022). Children are often compared to age-matched normally hearing controls (Bisconti et al., 2016; Bell et al., 2020) and in few cases with adults (Wu et al., 2024, 2025). While most studies were cross-sectional, especially in the case of few-month-old infants (Liu et al., 2023; Tan et al., 2024), given the logistical complexity of neonatal follow-up, a few included short-term prospective follow-up after implantation, within a 3-to-36 (Wu et al., 2024, 2025) month window (Wang et al., 2021; Esmaelpoor et al., 2025).

In adults, fNIRS has been applied to post-lingually deaf CI users to explore plasticity and speech comprehension (Bisconti et al., 2016; Chen et al., 2016; Zhou et al., 2018; Sherafati et al., 2022), as well as to assess pre-implantation predictors of CI outcomes (Anderson et al., 2019; Esmaelpoor et al., 2025). These studies were predominantly cross-sectional, though some included pre/post comparisons (Huang W. et al., 2025).

#### 4.1.2 Research objectives

The principal aims of the reviewed studies fall into the following categories: (1) characterizing cortical activation patterns in response to auditory and audiovisual stimuli in CI users, often comparing them to normal-hearing controls (Chen et al., 2016, 2017; Zhou et al., 2018; Wang et al., 2021, 2022; Coëz et al., 2022; Sherafati et al., 2022; Steinmetzger et al., 2022; Wu et al., 2024); (2) assessing cross-modal plasticity, particularly visual takeover of the auditory cortex in deaf individuals and CI users (Dewey and Hartley, 2015; Zhou et al., 2018, 2023; Bell et al., 2020; Shader et al., 2022; Liu et al., 2023, 2025; Chen et al., 2024; Deroche et al., 2024; Tan et al., 2024); (3) evaluating predictors of cochlear implantation outcomes, where preoperative fNIRS patterns have been shown to correlate with post-implantation speech perception (Anderson et al., 2019; Chen et al., 2022, 2024; Zhou et al., 2023; Esmaelpoor et al., 2025; Huang X. et al., 2025); (4) studying listening effort and cognitive load, especially in children and older adults under varying signal-to-noise conditions (Rovetti et al., 2019; Wang et al., 2025); and (5) exploring brain signal variability as an index of auditory-cognitive engagement in older adults (Rovetti et al., 2019; Mai et al., 2024; Lee et al., 2025; Wang et al., 2025).

#### 4.1.3 Main findings

Studies consistently showed that fNIRS could detect meaningful differences in cortical responses between hearing-impaired and normal-hearing populations. In CI users, increased activation in frontal and prefrontal areas—particularly the dorsolateral prefrontal cortex—has been interpreted as compensatory cognitive effort during speech perception supporting auditory processing (Sherafati et al., 2022). These findings were especially prominent when stimuli were presented in noisy environments, pointing to an increased listening effort in these individuals. In children, greater activation in the inferior temporal and frontal regions (frontal gyrus) has been associated with better auditory performance, even in noisy environments (Zhou et al., 2023; Balint et al., 2024).

In specific clinical populations, such as children with congenital toxoplasmosis, fNIRS revealed auditory cortex activation patterns suggestive of early functional reorganization even when behavioral thresholds were normal (Bertachini et al., 2021). Early reorganization was also found in 3 to-10 month-old infants with single-sided deafness (SSD), who showed increased functional connectivity in the right hemisphere at rest (Liu et al., 2023), and in 3 to 9 month-old infants with congenital hearing loss, who presented reduced network asymmetry and efficiency compared to normal hearing controls (Liu et al., 2025).

Older children predominantly underwent passive listening tasks with different auditory stimuli. For instance, both (Wang et al. 2022) and (Wu et al. 2024) used speech, music, and noise. However, while (Wang et al. 2022) investigated the influence of the implantation side on hearing abilities, (Wu et al. 2024) studied cortical network development. (Wang et al. 2022) findings suggest that left-sided CI children present better discrimination abilities of non-speech sounds, but no different hemodynamic patterns to speech stimuli compared to right-sided CI children (Wang et al., 2022). (Wu et al. 2024) results showed that the left temporo-fronto-parietal network develops faster than its right counterpart in CI children (Wu et al., 2024). Other studies used vocal/speech-only stimuli. (Wang et al. 2021) presented vocal emotional stimuli in toddlers with CIs, and found activation differences in the superior and middle temporal gyri before and after cochlear implantation. (Chen et al. 2022) presented CI children with sentences with weak vs. strong prosodic patterns (i.e., regularly pronounced vs. rhyming words with a strong rhythm), who showed less activation for the first compared to the latter, but an overall reduced activation compared to normal hearing control children. A few studies tested audiovisual integration of speech stimuli (Mushtaq et al., 2020; Alemi et al., 2023b; Zhou et al., 2023), in general supporting the idea of adaptive cross-modal plasticity in developmental populations. Lastly, one study (Alemi et al., 2023a) even looked at motor cortex co-activation in CI children, with no significant differences in somatosensory cortical activation between CI children and normal hearing peers. In general, although a standardized procedure has not yet been established and many different experimental paradigms and setup have been used (see also Supplementary Table 1), fNIRS demonstrated considerable flexibility in its application to developmental clinical populations, opening possibilities for the development of objective screening tools for preverbal or non-cooperative populations.

At the opposite end, in elderly individuals, reduced brain signal variability has been interpreted as a marker of decreased neural flexibility, particularly under increased auditory load (Wang et al., 2025). Some studies have shown that fNIRS changes in hemodynamic response amplitude with sound level track subjective perceptions, suggesting potential applications for hearing aid optimization in older adults (Sheffield et al., 2023), especially those with age-related hearing loss. Additionally, fNIRS has revealed diminished brain signal variability during cognitively demanding listening tasks. This has been interpreted as a marker of reduced neural adaptability and might help explain difficulties in complex listening environments.

Prospective studies with short-term follow-up (3–6 months) have started to validate the prognostic utility of fNIRS in identifying individuals at risk for poor CI outcomes (Anderson et al., 2019; Zhou et al., 2023). Longitudinal investigation up to 32 months in toddlers revealed that cortical responses in the left anterior temporal lobe changed with increasing hearing experiences in an oscillatory manner, with speech and noise processing developing in opposite phases. Importantly, these changes were also capable of predicting post-implantation improvement on auditory and communication performance (Wu et al., 2024). Additional longitudinal data (up to 36 months) revealed non-linear trajectories for the majority of developing functional connections of the speech-processing network with increasing CI experiences. In addition, development of the speech-processing network for both resting state and speech listening was predictive of later auditory performance for the CI children (Wu et al., 2025). The use of different stimuli (more than 2) has also been proven as a valuable tool to predict the development of auditory and verbal communication skills following CI, via machine learning models, even more accurately than the collection of audiological and demographic parameters (Chen et al., 2024).

Studies also indicate that fNIRS could be used to study binaural integration and interaural processing strategies (Laurent et al., 2021) and cross-modal activation of the auditory cortex by visual stimuli (Dewey and Hartley, 2015; Chen et al., 2016; Zhou et al., 2022, 2023). While some studies suggest a detrimental effect of cross-modal activation on CI performance (Dewey and Hartley, 2015; Zhou et al., 2022), others argued for a compensatory or even beneficial role, especially when visual speech cues were involved (Chen et al., 2016, 2017; Zhou et al., 2023; Deroche et al., 2024). Less is known about not-profound hearing loss, but increased activation was documented in frontal areas to visual speech in participants with partial hearing loss, and frontal activation in medial, middle, and inferior frontal gyrus was correlated with an increased visual bias in an audiovisual speech task. But the available literature did not report evidence for visual cross-modal recruitment of auditory cortex in this specific type of subjects (Aguiar et al., 2025).

Rare comparisons are available between fMRI and fNIRS results in CI patients (Sevy et al., 2010) and about less than profound hearing loss, and few studies investigated the presence of cross-modal responses (Puschmann and Thiel, 2017; Rosemann and Thiel, 2018, 2020).

#### 4.1.4 Clinical implications

The findings presented above highlight that fNIRS is already extensively used in hearing research and that it provides new insights into how hearing aids and CIs influence the development of neural structures across different stages of life, including both children and adults.

Moreover, fNIRS has been used to assess cortical activation in relation to the speech perception in challenging environments (Zhou et al., 2023), to visual stimuli (Dewey and Hartley, 2015; Chen et al., 2016), and to specific etiologies of hearing loss (Bertachini et al., 2021) in children.

In the elderly, fNIRS has demonstrated how the brain reacts under high auditory load (Wang et al., 2025), its potential role in optimizing hearing aids fitting (Sheffield et al., 2023), and its use in evaluating processing strategies in CI users (Steinmetzger et al., 2022), even among those with poor outcomes (Anderson et al., 2019; Zhou et al., 2023).

Importantly, the reviewed studies differ in sample sizes (both numerically and in audiological characteristics), fNIRS montages and system characteristics, experimental designs, procedures and stimuli. While these methodological choices should be interpreted in light of differing research questions, standardization is required to enable translation of these findings into clinical applications.

The few comparisons between fNIRS and fMRI sustain the finding of similar results.

Overall, this research may have a significant impact on the clinical management of patients with hearing loss, making fNIRS one of the most promising tools in this field.

### 4.2 fNIRS applications in tinnitus

Tinnitus is a multifaceted auditory condition characterized by the perception of phantom sounds in the absence of external input (San Juan et al., 2017). It varies in intensity and qualitative perception, often impairing quality of life and being associated with symptoms such as anxiety, sleep disturbances, and depression (Hu et al., 2024). Tinnitus is increasingly recognized as a complex neurophysiological phenomenon often accompanied by a range of comorbidities. Recent clinical studies have confirmed a high prevalence of conditions such as atherosclerosis (33.3%), hypertension (32.7%), and dyslipidemia (30.6%) in tinnitus patients, underscoring the systemic nature of the condition (Maihoub et al., 2025). Despite its high prevalence and considerable burden, the pathophysiology of tinnitus remains poorly understood, and its clinical management continues to present significant challenges (San Juan et al., 2021).

In recent years, fNIRS has emerged as a promising neuroimaging technique for elucidating the cortical mechanisms of tinnitus, largely for the same reasons described in the context for hearing loss (see the previous paragraph).

#### 4.2.1 Study population and design

The included studies (Supplementary Table 2) demonstrated significant heterogeneity in experimental design and sample characteristics. The number of participants ranged from approximately 10 (Schecklmann et al., 2014) to over 40 (Yu et al., 2023; Fan et al., 2024), predominantly involving individuals with chronic subjective tinnitus. Some studies included healthy control groups (Issa et al., 2016; Shoushtarian et al., 2024; Martins et al., 2025), while many reported sex imbalances and did not control for handedness, a variable potentially relevant to cortical lateralization processes (Fan et al., 2024; Martins et al., 2025).

Participants' age ranged from 18 to 80 years, with no systematic stratification of results by age groups, despite age being known to affect brain plasticity and connectivity (Issa et al., 2016; Fan et al., 2024).

From a methodological standpoint, the reviewed studies employed diverse experimental paradigms, highlighting the versatility of fNIRS. Some used auditory stimuli - such as notch-filtered sounds or white noise-to investigate both auditory plasticity and tinnitus masking mechanisms (Sun et al., 2020; Huang et al., 2021). Others focused on the effects of therapeutic interventions, including acupuncture, transcranial direct current stimulation (tDCS), repetitive transcranial magnetic stimulation (rTMS), and CIs, examining their impact on cortical activity (Schecklmann et al., 2014; Verma et al., 2019; Yu et al., 2023; Fan et al., 2024; Shoushtarian et al., 2024).

Additional studies were conducted under resting-state conditions, focusing on functional connectivity between auditory and non-auditory brain regions (San Juan et al., 2017, 2021). Interestingly, some others introduced innovative experimental configurations, such as custom-designed probes and patient classification algorithms (San Juan et al., 2021; Shoushtarian et al., 2024).

#### 4.2.2 Main findings

One of the most consistent findings across studies has been the increased activity in the superior temporal regions, particularly the primary auditory cortex and superior temporal gyrus. This has been interpreted as a form of maladaptive plasticity of the auditory system, possibly due to partial deafferentation (Sun et al., 2020; Huang et al., 2021).

Beyond sensory areas, significant activation has also been observed in frontal regions—particularly the dorsolateral prefrontal cortex and anterior cingulate cortex—which are implicated in cognitive and attentional processes. These activations were heightened during attentional tasks or in response to therapeutic interventions, suggesting that these regions play an active role in the persistence of tinnitus perception and were closely linked to mechanisms of selective attention and cognitive control (Shoushtarian et al., 2024; Martins et al., 2025).

Altered connectivity between auditory areas and limbic structures such as the amygdala and medial prefrontal cortex has been frequently reported, particularly in patients experiencing high levels of anxiety or stress. These findings supported the hypothesis that tinnitus involved a dysfunctional network in which internal auditory signals were misinterpreted as relevant or threatening (Issa et al., 2016; San Juan et al., 2017, 2021).

From a therapeutic viewpoint, acupuncture was associated with a subjective reduction in tinnitus distress and increased prefrontal activation (Yu et al., 2023; Fan et al., 2024), while tDCS and rTMS induced more localized cortical changes, with potential implications for targeted neuromodulation strategies (Schecklmann et al., 2014; Verma et al., 2019).

These interventions appeared to modulate cortical activity through distinct mechanisms: tDCS alters neuronal excitability depending on the applied polarity (Verma et al., 2019), whereas rTMS induces long-lasting plastic changes, especially in the frontal cortex (Schecklmann et al., 2014). Acupuncture has been shown to activate the auditory cortex and increase oxygenated hemoglobin (HbO_2_) levels in the temporal lobe, suggesting a direct effect on neural circuits underlying auditory perception in patients with tinnitus (Yu et al., 2023).

Across all these applications, fNIRS enabled real-time monitoring of cortical hemodynamic changes, revealing significant increases in HbO_2_, particularly in the dorsolateral prefrontal cortex and temporal areas. CIs, lastly, have been found to affect the dynamics of the default mode network, suggesting a widespread reorganization of the involved cortical networks (Shoushtarian et al., 2024).

Concerning fMRI data, a greater activation of the auditory cortex was detected, with different patterns between patients with and without hyperacusis. Data about the Heschl's Gyrus are still controversial in relationship with the hippocampus activation, as well as those of the inferior colliculus. Other neural structures that can be investigated by fMRI but not by fNIRS may also play a role, such as the cochlear nuclei, thalamus, limbic system and cerebellum (Jimoh et al., 2023).

#### 4.2.3 Clinical implications

The collected evidence suggests that fNIRS may represent a promising tool for the functional assessment of tinnitus and for monitoring treatment effects. The observed activation in frontal regions and in deeper layers of the auditory cortex supports a multidimensional clinical approach that integrates auditory, cognitive, and emotional aspects (Martins et al., 2025). However, the current sample sizes are too limited in order to generalize these findings. Larger cohorts of tinnitus patients should be assessed with fNIRS, and several research protocols have already highlighted the need for this (Hu et al., 2024; Jiang et al., 2025; Huang X. et al., 2025). Once completed, these randomized controlled trials studies could provide valuable insights into the neural mechanisms involved in tinnitus and the potential efficacy of acupuncture treatment for tinnitus patients. Overall, the available fNIRS results appear to well replicate those obtained with fMRI. However, the main advantages of fNIRS over fMRI lie in its lower costs, which in turn might support broader clinical use, and its nearly silent acquisition, an important feature both for this clinical population and for ensuring better control over auditory stimuli. To the best of our knowledge, a direct comparison between the two techniques has not yet been conducted in tinnitus patients. Such a study would help establish standardized fNIRS acquisition procedures, which are essential for clinical implementation. Lastly, integration with artificial intelligence algorithms may pave the way for personalized therapy and neurofunctional patient classification (Shoushtarian et al., 2024).

### 4.3 fNIRS applications in vestibular research

The cortical processing of vestibular stimuli in humans is a complex and evolving area of investigation. While early research proposed the existence of a discrete “vestibular cortex,” current evidence favors a distributed cortical network involving mesial temporal, parietal, occipital, and frontal regions (Kahane et al., 2003; Duque-Parra, 2004; Arvaniti et al., 2024).

The use of fNIRS in vestibular settings has emerged as a valuable tool due to its portability and versatility in clinical environments, allowing the assessment of patients in various body positions or during standardized motion stimuli commonly used for balance assessment and also to study the possible correlation with psychosocial burden (Molnár et al., 2022).

#### 4.3.1 Study populations and design

In the reviewed studies (Supplementary Table 3), the number of participants ranged from approximately 5 (Iida et al., 2009) to 38 (Zhao et al., 2023), with an average sample size of 17 patients, predominantly aged between 18 and 65 years. Four studies explicitly included only right-handed participants (Karim H. T. et al., 2013; Takakura et al., 2015; Nguyen et al., 2020), while eight studies ensured sex balance (Iida et al., 2009; Karim H. et al., 2013; Hoppes et al., 2018a,b; Nguyen et al., 2020; He and Bao, 2024), and five included only male subjects (Kobayashi and Cheung, 2006; Iida et al., 2009; Karim H. T. et al., 2013; Takakura et al., 2015).

The majority of studies considered healthy subjects, with only one study focusing on individuals with visual vertigo (Hoppes et al., 2018a).

#### 4.3.2 Main findings

A diverse array of vestibular stimulation techniques was employed, among those commonly used in clinical practice to test balance function. These methods were designed to selectively activate specific components of the vestibular system, allowing clinicians to assess its integrity and contribution to postural control and spatial orientation. Applied techniques include head movements (Kobayashi and Cheung, 2006; He and Bao, 2024), caloric testing (Iida et al., 2009; Karim H. T. et al., 2013), rotary chair stimulation (Nguyen et al., 2020), dynamic posturography (Karim H. T. et al., 2013), motion platforms (Zhao et al., 2023), subjective visual vertical tasks (Lu et al., 2024), galvanic vestibular stimulation (GVS) (Valdés et al., 2021; Hernández-Román et al., 2023), and optic flow in virtual reality environments (Hoppes et al., 2018a,b).

Studies employing head-movement paradigms reported significant increases in occipital HbO_2_ levels when participants moved their heads along the pitch axis, particularly with eyes open. (Kobayashi and Cheung 2006) found that visual-vestibular interactions during head rotation induced cortical activation in the right middle/inferior temporal gyri, bilateral supramarginal gyri, and superior temporal areas, with activity modulated by head velocity and perceptual mislocalization effects (Kobayashi and Cheung, 2006).

Caloric testing studies showed that warm water stimulation (44 °C) increased bilateral activation with ipsilateral dominance, while cold water (30 °C) decreased ipsilateral HbO_2_ signals (Iida et al., 2009). These findings suggested that cortical activation patterns are sensitive to both the temperature and the laterality of the stimulus. Karim H. T. et al. (2013) confirmed the temperature- and side-dependent cortical responses and noted age-related differences, with older adults showing broader bilateral activation (Karim H. et al., 2013).

Multisensory conflict paradigms combining rotary vestibular and visual stimuli demonstrated increased hemodynamic activity in the temporoparietal junction, medial temporal gyrus, and medial superior temporal area during incongruent stimulation (Nguyen et al., 2020). Activity in the supramarginal gyrus inversely correlated with perceived vertigo intensity, suggesting a role in sensory integration.

Dynamic posturography protocols disrupted somatosensory and visual inputs, promoting reliance on vestibular cues. These tasks consistently evoked bilateral activation in temporal-parietal regions, including the supramarginal and superior temporal gyri (Takakura et al., 2015). (Takakura et al. 2015) further implicated a right-hemispheric peri-Sylvian network for sensory synergy and a parieto-frontal network for postural command integration.

(Zhao et al. 2023) used a motion platform to induce whole-body translations and observed the most widespread cortical activation during circular motion, involving the postcentral gyrus, occipital cortex, temporoparietal junction, superior temporal gyrus, and bilateral prefrontal areas. Linear motions activated fewer areas (Zhao et al., 2023).

Subjective visual vertical tasks examined age-related differences in cortical response. (Lu et al. 2024) reported greater activation in bilateral postcentral gyri and right middle frontal gyrus in older adults, while young adults showed more lateralized responses. Cortical activation patterns correlated with task performance (Lu et al., 2024).

GVS was associated with robust cortical responses (Valdés et al., 2021), with greater deoxygenated hemoglobin in the left supramarginal gyrus during the test. (Hernández-Román et al. 2023) found activation in bilateral parietal and ipsilateral temporal cortices, mirroring those seen during passive movement.

Finally, optic flow paradigms revealed bilateral fronto-temporo-parietal activation in healthy individuals exposed to unidirectional visual motion, with and without fixation (Hoppes et al., 2018b). In patients with visual vertigo, a distinct pattern of reduced middle frontal activity was observed during optic flow, suggesting disrupted visual-vestibular interaction (Hoppes et al., 2018a).

fMRI has been used to study activation patterns in cortical and subcortical regions involved in balance and spatial orientation, such as the parietal operculum, insula, cerebellum, and vestibular cortex (Musat et al., 2025).

#### 4.3.3 Clinical implications

The reviewed studies highlight the versatility of fNIRS in capturing vestibular-related cortical activation across a broad range of stimulation modalities. This opens avenues for assessing sensorimotor integration, balance control, and visual-vestibular conflict. The studies described above should primarily be interpreted as pioneering and descriptive in terms of cortical activation, with clinical applications not yet established. They also pave the way for further applications of functional NIRS in clinical vestibular research, neurorehabilitation, and cognitive neuroscience. These fields, indeed, are currently lacking systematic and standardized research protocols applying fNIRS to patients with vestibular disorders. Although vestibular symptoms can arise from a variety of clinical conditions, fNIRS measurements can provide valuable insights into the underlying neural complexity and offer a more fine-grained understanding of these conditions.

Moreover, vestibular disorders can arise from a variety of clinical conditions which may significantly impact the quality of life (Cao et al., 2022; Molnár et al., 2022) and, sometimes, the co-occurrence of psychiatric symptoms as depression should be alerted. Therefore, fNIRS measurements can provide valuable insights into the underlying neural complexity and offer a more fine-grained understanding of these conditions. Furthermore, fNIRS is portable, well-tolerated, and suitable for repeated measures, enabling longitudinal monitoring of neural reorganization following rehabilitation or compensation. A systematic comparison of neuroimaging studies focussed on the assessment of consistent activation and deactivation among healthy participants and patients reporting vertigo (Varangot-Reille et al., 2022). The analysis included both fMRI and PET, but also the fNIRS. It confirmed that the vertigo seems to involve an activation of a cluster of structures that is consistent amongst the experiments and stimuli: the insula (anterior to posterior), the anterior cingulate cortex, the inferior parietal lobule, the putamen, the thalamus, the cerebellum, the precentral gyrus, and the superior temporal gyrus. Thus, it suggests the involvement of multiple vestibular and non-specific networks with the inclusion of a cortico–basal ganglia– cerebellar–thalamic network. Furthermore, the fMRI and related neuroimaging techniques confirmed a high spatial resolution, but low temporal resolution, so they could not propose any activation sequence (Luck, 2014; Varangot-Reille et al., 2022). On the other hand, fNIRS offers better temporal resolution among the hemodynamic techniques and can be applied in naturalistic conditions, making it a strong candidate for studying cortical involvement and reorganization in vestibular disorders.

## 5 General discussion

fNIRS represents a valuable neuroimaging modality for investigating cortical responses related to auditory and vestibular processing.

The clinical potential of fNIRS lies primarily in its ability to provide objective, ecologically valid, and device-compatible measures of cortical activity.

The present study aims to provide an overview of the current applications in audiological research, providing a general picture of these topics. While several reviews on fNIRS and hearing are already available in the literature, they tend to focus narrowly on single aspects, such as speech perception in CI users, pediatric applications, or methodological considerations regarding signal acquisition. Few have offered an integrative perspective that combines pediatric, adult, and elderly populations, covering the full spectrum from normal hearing to profound deafness and across multiple clinical and research contexts, such as hearing impairment, tinnitus and vestibular disorders.

This review supports the hypothesis that fNIRS can successfully capture neural activity associated with auditory perception in patients with hearing impairment, revealing patterns of plasticity and functional reorganization after the rehabilitation with hearing aids (Aguiar et al., 2025) and Cis (Chen et al., 2016, 2017; Zhou et al., 2018; Wang et al., 2021, 2022; Coëz et al., 2022; Sherafati et al., 2022; Steinmetzger et al., 2022; Wu et al., 2024). Its potential relies in being unharmful for patients and being minimally influenced by the CI use, thus making it available for longitudinal studies on the brain development, to assess common and different features compared to healthy subjects or to prove the presence of cross-modal development. It may offer added value in preoperative evaluations of CI candidates, especially when behavioral testing is limited or inconclusive. Moreover, its sensitivity to plasticity-related cortical patterns could guide individualized rehabilitation strategies, particularly in pediatric patients where early intervention is critical, allowing for an objective evaluation of outcomes. In addition, the abovementioned studies report the possible role using the fNIRS in early phases post implantation in predicting the long term outcome of patients (Chen et al., 2024).

In tinnitus, fNIRS has provided insight into both auditory and non-auditory cortical involvement and has proven sensitive to changes following various interventions, including neuromodulation and acupuncture (Schecklmann et al., 2014; Verma et al., 2019; Yu et al., 2023; Fan et al., 2024; Shoushtarian et al., 2024). Although further clinical validation and methodological standardization are needed, fNIRS may become a valuable resource for integrating the perceptual, affective, and cognitive components of tinnitus into a more comprehensive and personalized clinical and therapeutic framework.

Applications in vestibular clinical settings remain limited, but fNIRS seems useful for unveiling mechanisms of cortical balance processing, even in real time during vestibular stimulation mapping of multisensory integration and balance-related cortical networks under both static and dynamic stimulation conditions. While only one study involved patients with visual vertigo (Hoppes et al., 2018a), the others investigate vestibular function basically in healthy subjects. Its ability to capture cortical activity during movement and in ecologically valid conditions makes it particularly valuable for vestibular research, where traditional imaging methods are often constrained. By integrating fNIRS with other modalities, researchers can achieve a more comprehensive understanding of the cortical mechanisms underlying balance control.

At present, fNIRS does not replace more established neuroimaging techniques but serves as a complementary tool, having undeniable advantages and disadvantages compared to fMRI or PET.

Indeed, despite the promising applications, several limitations must be acknowledged. fNIRS's relatively low spatial resolution, limited penetration depth (restricted largely to superficial cortical layers), susceptibility to motion artifacts, and limited temporal resolution may constrain interpretation, especially for rapid sensory events. Inter-individual variability in scalp-brain coupling and anatomical landmarks adds further complexity.

Moreover, most current studies are cross-sectional and small-scale, limiting generalizability. While a few longitudinal or pre/post designs exist, larger prospective trials with long-term follow-up are needed to validate prognostic applications. Additionally, standardized protocols and control groups remain insufficient.

To address these limitations, future research should prioritize multicenter studies with harmonized protocols, longitudinal designs to track neural plasticity over time, and multimodal approaches combining fNIRS with other neuroimaging techniques-such as EEG or fMRI-for a more comprehensive characterization of the cortical networks involved (Shoushtarian et al., 2024; Martins et al., 2025).
