# Supplementary material for: A narrative review of functional near-infrared spectroscopy (fNIRS) applications in hearing loss, tinnitus and vestibular disorders
Source: Front Neurosci. 2026 Jan 23;19:1703917. doi: 10.3389/fnins.2025.1703917 (PMC12876175; doi:10.3389/fnins.2025.1703917)
Supplement: Supplementary file 1 [file Data_Sheet_1.pdf]

Supplementary Material

1 Supplementary Tables

Supplementary Table 1. Summary table of included papers in the hearing disorder section.
Abbreviations:
Study population: AoI – Age of Implant; CI – Cochlear Implant; HA – Hearing Aid; HL - Hearing Loss; SNR – Signal to Noise Ratio; SRT – Speech Reception Threshold; SSD – Single-sided Deafness.
Comparison control group: NH – Normal Hearing.
fNIRS Characteristics: IFG – Inferior Frontal Gyrus; IPL – Inferior Parietal Lobes; ROI – Region of Interest; STG – Superior Temporal Gyrus.

Paper	Study Population (age)	Comparison control group (age)	fNIRS characteristics	Stimuli/Experimental Paradigm
Studies in adult populations				
(Anderson *et al.*, 2019)	17 adults (58 years, SD=13.9) bilateral profound HL unilateral CI	17 NH controls (57 years, SD=16.8)	24 channels Occipital, parietal, and right prefrontal cortices Hitachi ETG-4000 system	Silent visual speech (lipreading)
(Balint *et al.*, 2024)	34 CI user: 22 with SRT < 10 dB SNR (53±19 years) 12 with SRT > 10 dB SNR (56±15 years)	22 NH controls (31±69 years), mean SRT = -6.1 dB SNR	40 channels Bilateral STG, left prefrontal cortex (Broca's area) Shimadzu FOIRE-3000 system	Auditory: speech sentences in quiet and noise, with 4-choice comprehension task
(Bisconti *et al.*, 2016)	10 CI users (52.7 ± 17.3 years) 2 prelingual, 8 postlingual	10 NH controls (50.6 ± 17.2 years)	32 channels Frontal, temporal and parietal regions TechEn's CW6 system	Listening tasks: active (phonological awareness) and passive (passages comprehension)
(Chen *et al.*, 2016)	20 postlingual CI users (54.58 ± 14.96 years)	20 NH controls (54.89 ± 15.80 years)	20 channels ROIs: bilateral auditory and visual regions	Auditory: word stimuli Visual: checkerboard

			NIRx NIRScout system	
(Chen *et al.*, 2017)	19 postlingual deaf adults with unilateral CI<br><br>(54.58 years, SD=14.96)	19 NH (54.89 years, SD=15.80)	Simultaneous EEG and fNIRS acquisition<br><br>fNIRS: 20 channels Bilateral auditory and visual areas<br><br>NIRx NIRScout system<br><br>EEG: 96 channels BrainProducts	Auditory: 1 kHz pure tone<br>Visual: circular checkerboard patterns
(Dewey and Hartley, 2015)	30 candidates for CI with profound HL<br>(41 ± 11 years)	30 NH controls (34 ± 13 years)	24 channels Bilateral auditory cortex<br>Hitachi ETG-4000 system	Auditory: unmodulated and amplitude-modulated noise<br>Visual stimuli: randomly and coherently moving dots<br>Somatosensory: 10 and 20 Hz vibrotactile stimulation
(Esmaelpoor *et al.*, 2025)	27 postlingual deaf adults with CI (58.1 ± 19.3 years)	No	52 channels<br>ROIs: bilateral temporal lobes, left prefrontal and occipital regions<br>NIRx NIRScout system	Resting-state recordings - 4 sessions (pre-CI, 1m, 3m, 12m)
(Huang *et al.*, 2025)	26 postlingual deaf adults (48.4 ± 20.4 years)<br>9 CI group<br>17 no-surgery group	No	44 channels<br>Temporal and occipital regions<br>Hitachi ETG-4000 system	Resting state
(Laurent *et al.*, 2021)	19 bilateral deaf adults	18 NH controls (26.1 years, SD=6.6)	52 channels<br>ROIs: frontal, central and occipital areas	Decision-making task with

	(28.1 years, SD=8.8)		NIRX NIRScout system	spatiotemporal inferences
(Lee *et al.*, 2025)	63 adults with bilateral moderate or severe HL<br>49 cognitive normal group (73.6±7.5 years)<br>14 MCI at risk group<br><br>(74.7±6.8 years)	No	48 channels<br><br>Bilateral prefrontal cortex and orbitofrontal cortex OBELAB NIRSIT system	Cognitive tasks: verbal fluency, picture naming, verbal working memory, delayed verbal retrieval and STROOP task.
(Mai *et al.*, 2024)	10 healthy ageing adults with high-frequency mild-to-moderate HL (70 years, SD=4.5)	No	44 channels<br>Temporal, parietal and frontal regions<br>Hitachi ETG-4000 system	Auditory: speech and non-speech stimuli<br>Visual: checkerboard<br>fNIRS before and after speech in noise training at home
(Rovetti *et al.*, 2019)	16 HA users (72.06 years, SD = 6.63)	No	16 channels<br>Prefrontal cortex<br>fNIR Imager 1100 system	Working memory tasks: verbal n-back task with visual and auditory stimuli with and without HA.
(Shader, Luke and McKay, 2022)	12 postlingual deaf adults with CI (62.3 years)	No	44 channels<br>Frontal, temporal and occipital lobes<br>NIRX NIRScout system	Auditory: story audio segments<br>Visual: same story video segments without audio
(Sheffield *et al.*, 2023)	13 bilateral CI users<br>(49.1 years)	16 NH controls (50.1 years)	22 channels<br>Left fronto-temporal region<br>Hitachi ETG-4000 system	Auditory: signal-correlated noise
(Sherafati *et al.*, 2022)	20 postlingual deaf adults with unilateral right-sided CI<br>(56.80 years, SD=14.09)	18 NH controls (57.57 years, SD=12.74)	High-density diffuse optical tomography (HD-DOT, Eggebrecht et al., 2014)<br>ROIs: Left	Spoken word recognition paradigm

			prefrontal cortex, left and right auditory regions	
(Steinmetzger *et al.*, 2022)	20 adults with SSD and unilateral CI (60 years)	No	Simultaneous EEG and fNIRS acquisition<br><br>fNIRS: 40 channels Bilateral auditory cortex NIRx NIRScout system<br><br>EEG: 64 channels BrainVision actiCHamp system	Auditory: German vowels with fixed or variable prosody
(Wang *et al.*, 2025)	25 mild HL (66.0 ± 3.7 years) 26 moderate to severe HL (67.5 ± 3.7 years)	30 NH controls (65.5± 3.4 years)	63 channels Temporal, parietal, and frontal regions NirScan-9000A system	Resting state Auditory task: sentences in quiet, +5 dB SNR and 0 dB SNR
(Zhou *et al.*, 2018)	15 CI users (64.2 ±10.1 years)	14 NH controls (53.5±12.0 years)	46 channels Prefrontal cortex NIRx NIRScout system	Resting state Word recognition task: auditory-only and visual-only (lipreading) words
		Studies in developmental populations		
(Alemi, Wolfe, Neumann, Manning, Towler, *et al.*, 2023)	50 children with CI (7-18 years) Implanted before age 4 26 Typical Language group 24 Low Language group	25 NH children (7-18 years)	Non-simultaneous EEG and fNIRS acquisition<br><br>fNIRS: 122 channels<br><br>NIRx NIRScout system<br><br>EEG: 128 EGI system	Audio-visual integration task: syllables presented in visual-only, auditory-only, and auditory-visual modes

(Alemi, Wolfe, Neumann, Manning, Hanna, *et al.*, 2023)	50 children with CI (7-18 years) Implanted before age 4<br>26 Typical Language group<br><br>24 Low Language group	25 NH children (7-18 years)	122 channels<br><br>NIRx NIRScout system	Motor task: squeezing and resting
(Bell *et al.*, 2020)	15 children with bilateral HL (9.23 years, SD=1.93), with bilateral HA;<br>20 children with ADHD (10.25 years, SD=2.01)	27 typically developing children (9.87 years, SD=1.77)	44 channels<br>Bilateral frontal, temporal and parietal regions<br>ETC4000 Hitachi system	Go/no-go task with auditory (sinusoidal tones of varying frequencies) and visual (letters) stimuli
(Bertachini *et al.*, 2021)	38 infants with congenital toxoplasmosis (58 days, SD= 22 days)	23 healthy infants (56 days, SD= 21)	70 channels<br>Frontal, temporal, parietal lobes<br>NIRx NIRScout system	Auditory: mother and researcher infant directed speech, mother and researcher adult directed speech
(Chen *et al.*, 2022)	21 HL prelingual deaf children with unilateral right-sided CI (6;8 years, range 5;0–10;9)<br>AoI: 3;1 (1;5–9;7)	25 NH children (6;0 years, range 5;1–7;8)	20 channels<br>Bilateral temporal, inferior frontal, and inferior parietal cortices<br>LIGHTNIRS	Auditory: strong-prosodic vs. weak-prosodic sentences
(Chen *et al.*, 2024)	47 children with severe-to-profound HL and unilateral CI (35.47 ± 17.24 months)<br>AoI: 12– 48 months	No	20 channels<br>Bilateral auditory and language-related areas<br>System not specified	Auditory: speech, noise, speech in noise, and music
(Coëz *et al.*, 2022)	9 children (370 days) with profound HL and	8 NH children (370 days)	30 channels<br>Auditory areas in the bilateral temporal, frontal	Auditory: voice and non- voice stimuli

	HA, candidates for CI		and parietal areas NIRx NIRScout system	
(Deroche *et al.*, 2024)	50 children with CI (7-18 years) Implanted before age 4 26 Typical Language group 24 Low Language group	25 NH children (7-18 years)	Non-simultaneous EEG and fNIRS acquisition<br><br>fNIRS: 122 channels<br><br>NIRx NIRScout system<br><br>EEG: 128 EGI system	Visual: rotating circular checkerboard
(Liu *et al.*, 2025)	55 HL infants with congenital mild-to-profound HL (5.87 ± 1.89 months)	60 NH (5.91 ± 1.97 months)	64 channels Frontal, temporal, parietal, and occipital areas NirSmart system	Resting state
(Liu *et al.*, 2023)	36 infants with congenital HL: 18 SSD (4.55 ±1.60 months) 18 mild- to-moderate UHL (4.93 ± 1.78 months)	12 NH (4.93 ± 1.78 months)	64 channels Frontal, temporal, parietal, and occipital areas NirSmart system	Resting state
(Mushtaq *et al.*, 2020)	19 children (9.5 years) with congenital or early-onset HL and bilateral Cis AoI: 27.4 months (10– 86 months)	20 NH children (8.4 years)	24 channels Temporal areas Hitachi system	Visual: visual speech stimulus only (lip-reading) Auditory: normal speech, Signal-correlated noise (SCN), steady speech- shaped noise (SSSN)
(Sevy *et al.*, 2010)	37 HL children with CI > 4 months (7.9 ± 3.8 years) 9 HL children at	11 NH children (9.4 ± 3.4 years) 11 NH adults (30.4 ± 8.3 years)	4 channels Temporal lobe and STG TechEn NIRS 2CE system	Auditory: story sentences

	CI activation (4.7 ± 1.6 years)			
(Tan *et al.*, 2024)	22 HL infants (4 months) with congenital HL > 40 dB	28 NH newborns (1– 2 days)<br>15 NH infants (4 months)	36 channels<br>Frontal, temporal, and parietal cortices<br>NirSmart system	Resting state
(Wang *et al.*, 2021)	22 prelingual severe-to-profound HL infants<br>(22.5 ± 6.43 months)<br>tested Pre-CI with HA and Post-CI with Unilateral CI;<br>AoI: 22.5 months (13– 38)<br>contralateral HA	No	52 channels<br>Temporal, frontal, and central regions<br>NirSmart system	Auditory: pseudo-sentences with different emotional contents (fear, angry, happy, neutral)
(Wang *et al.*, 2022)	34 HL infants (36.82 ±17.65 months) with prelingual severe-to-profound HL and unilateral CI (15 left CI and 19 right CI)<br>AoI: 32.73 months (SD=15.43)	35 NH adults (22.8 ± 1.78 years)	20 channels<br>Temporal lobe and adjacent language processing areas<br>NIRx NIRSport2	Resting state<br>Auditory: speech, music, and noise
(Wu *et al.*, 2024)	67 HL children (3.02 ± 2.17 years) with prelingual severe-to-profound HL with CI (5 bilateral CI, the rest unilateral)<br>AoI: 33 months (SD=16)	35 NH adults (22.8±1.78 years)	20 channels<br>Bilateral temporo-parieto-frontal auditory and language-related areas<br>NIRx NIRSport2	Resting state<br>Auditory: speech, music, noise and speech in noise
(Wu *et al.*, 2025)	84 HL children (3.02 ± 2.17 years) with prelingual severe-	35 NH adults (22.8±1.78 years)	20 channels<br>Bilateral temporo-parieto-frontal auditory	Resting state<br>Auditory: speech, music, noise, and speech in noise

	to-profound HL with CI (6 bilateral CI, the rest unilateral) AoI: 38.75 months (25.59)		and language-related areas NIRx NIRSport2	
(Zhou *et al.*, 2023)	38 prelingually deaf children with CI (6.86 ± 0.7 years)	36 NH children (7.04 ± 0.89 years)	44 channels Bilateral STG & IPL, left IFG Hitachi ETG-4100 system	Auditory: speech in quiet and speech in noise Visual: silent visual speech (lip-reading) of video-sentences.

Supplementary Table 2. Summary table of included papers in tinnitus section.
Abbreviations:
Hearing condition: CI – Cochlear Implant; HL – Hearing Loss; NH – Normal Hearing.
fNIRS Characteristics: BA – Brodmann Area; ROI – Region of Interest; STC – Superior Temporal Sulcus.
Experimental Paradigm: BBN – Broadband Noise; WN – White Noise.

Paper	Study Population (mean age in years)	Hearing Condition	Control group	fNIRS Characteristics	Experimental Paradigm
(Fan *et al.*, 2024)	14 tinnitus patients (48±11.68)<br><br>14 controls (26.86±3.60)	no threshold reported	yes	20 channels<br>Bilateral temporal lobes<br>NirSmartII-3000C	Auditory - pink noise, 750 and 8000 Hz noise
(Huang *et al.*, 2021)	29 tinnitus patients (31.45 ± 9.68)	NH or mild HL	no	44 channels<br>ROIs: STC and BA64<br>Hitachi ETG-4000	Resting state before and after sound therapy
(Issa *et al.*, 2016)	10 tinnitus patients (48.7±16)<br><br>7 controls (25.7 ± 7.8)	NH	yes	44 channels<br>Primary auditory cortex and surrounding auditory belt regions<br>TechEn's CW6 system	Auditory - 750 or 8000 Hz tones, BBN
(Martins *et al.*, 2025)	23 tinnitus patients (NA)<br><br>23 controls (NA)	NH	yes	20 channels<br>Primary auditory cortex<br>NIRx NIRSport1	Auditory - WN, 1 kHz, and tinnitus frequency/10 kHz
(San Juan *et al.*, 2017)	10 tinnitus patients (48.7±16)	NH	yes	44 channels<br>Primary auditory cortex and surrounding	Auditory - pure tone or BBN and resting state

	8 controls (25.4±7.3)			temporal and parietal association cortices TechEn's CW6 system	before and after auditory stimuli
(San Juan *et al.*, 2021)	20 tinnitus patients (38.2)<br><br>20 controls (48)	NH	yes	46 channels Auditory cortex and surrounding temporal and parietal association cortices TechEn's CW6 system	Auditory - BBN and resting state before and after auditory stimuli
(Schecklmann *et al.*, 2014)	12 tinnitus patients (48.2 ± 10.7) - Verum group<br><br>11 tinnitus patients (46.5 ± 11.5) - Sham group<br><br>12 Healthy control group	NH	yes	44 channels Auditory cortex Hitachi ETG4000	Auditory - Speech Noise
(Shoushtarian *et al.*, 2024)	14 tinnitus patients with CI (65,7 ± 11)	HL with CI usage	no	34 channels ROIs: temporal cortices NIRx NIRScout	Resting state and evoked responses to auditory and visual stimulation
(Sun *et al.*, 2020)	13 tinnitus patients (37 ± 9)<br><br>20 controls (31 ± 6)	NH or mild HL for the tinnitus patients, NH for the controls	yes	44 channels Bilateral auditory cortex Hitachi ETG4000	Auditory - WN, BBN, notched sound 4, 8 kHz

(Verma, Jha and Singh, 2019)	1 tinnitus (28)	HL	no	20 channels Auditory cortex bilaterally NIRx NIRScout 8×8	Auditory - 3 seconds sounds
(Yu *et al.*, 2023)	18 tinnitus patients (49.38)	NH	no	20 channels NirSmartII-3000C	Auditory - 8 kHz tone

Supplementary Table 3. Summary table of included papers in the vestibular research section.
Abbreviations:
fNIRS Characteristics: BA – Brodmann Area; ROI – Region of Interest.

Paper	Study Population (mean age in years)	Control group	fNIRS characteristics	Stimuli/Experimental Paradigm
(He and Bao, 2024)	28 healthy subjects (25 ± 3)	no	24 channels<br>Bilateral temporo-parieto-occipital<br>LABNIRS system	Head-rotation-induced flash-lag effect
(Hernández-Román *et al.*, 2023)	18 healthy subjects (23 ± 0.6)	no	26 channels<br>Bilateral temporal and parietal cortices<br>NIRx NIRScout	Galvanic vestibular stimulation
(Hoppes *et al.*, 2018a)	15 Visual Vertigo patients (39 ± 12)<br>15 healthy controls (38 ± 12)	yes	32 channels<br>Bilateral temporal and occipital lobes<br>TechEn's CW6 system	Optic flow-virtual reality
(Hoppes *et al.*, 2018b)	15 healthy subjects (41)	no	32 channels<br>Bilateral fronto-temporo-parietal and occipital lobes<br>TechEn's CW6 system	Optic flow-virtual reality with and without a fixation target (2 testing sessions)
(Iida, Haida and Igarashi, 2009)	5 healthy subjects (25.8)	no	24 channels<br>Bilateral temporal region<br>Hitachi ETG-100 system	Caloric test

(H. Karim *et al.*, 2013)	15 healthy subjects (28 ± 9)	no	32 channels<br>Bilateral frontal, temporal and parietal cortices TechEn's CW6 system	Dynamic posturography with Sensory Organization Test protocol
(H. T. Karim *et al.*, 2013)	20 healthy subjects: 10 in the younger group (25 ± 6); 10 in the older group (74 ± 5)	no	30 channels<br>Bilateral prefrontal and temporal cortices TechEn's CW6 system	Caloric test
(Kobayashi and Cheung, 2006)	6 healthy subjects (30-50 years)	no	2 channels<br>1 Parietal (Pz), 1 Occipital (O2)<br>Two custom-built NIRO-300G spectrophotometers	Head movements in the pitch axis
(Lu *et al.*, 2024)	38 healthy subjects: 19 in the younger group (22.30 ± 2.05);<br>19 in the older group (68.37 ± 4.67)	no	41 channels<br>Bilateral prefrontal, primary sensory and motor, and occipital cortices<br>NirSmart-3000A	Subjective visual vertical task paradigm
(Nguyen *et al.*, 2020)	14 healthy subjects (25.8 ± 8.2)	no	44 channels<br>Bilateral temporo-parietal regions<br>LIGHTNIRS system	Rotatory chair
(Takakura *et al.*, 2015)	11 healthy subjects (33.4 ± 7.4)	no	50 channels<br>Right Hemisphere only<br>Shimadzu OMM-3000 system	Sensory Organization Test protocol

(Valdés *et al.*, 2021)	12 healthy subjects (29 ± 6)	no	12 channels<br>ROIs: BA40 and BA3<br>NIRx NIRSport 2 system	Noisy Galvanic vestibular stimulation
(Zhao *et al.*, 2023)	12 healthy subjects (24.5)	no	10 channels<br>Right sensorimotor and temporo-parietal cortex<br>Imagent ISS system	Motion platform: whole-body passive translational motion (circular, lateral, and fore-and-aft)
